# A Perfect Home

**Published:** 1874-12

**Authors:** 


A Perfect Home.—The most perfect home I ever saw was a little house into the sweet incense of whose fires went no costly things. A thousand dollars served as a year's living of father, mother and three children. But the mother was the creator of a home; her relations with her children were the most beautiful I have ever seen; even the dull and common-place man was lifted up and enabled to do good work for souls by the atmosphere which this woman created; every inmate of her house involuntarily looked into her face for the key-note of the day, and it always rang clear. From the rosebud or clover leaf, which in spite of her hard house-work she always found to put by our plates at breakfast, down to the story she had on hand to be read in the evening, there was no intermission of her influence. She has always been, and always will be, my ideal of a mother, wife and home-maker. If to her quick brain, loving heart and exquisite face had been added the appliance of wealth and the enlargements of wider culture, hers would have been absolutely the ideal home. As it was, it was the best I have ever seen.

